# Principles of Effective Simulation-Based Teaching Sessions in Medical Education: A Narrative Review

**DOI:** 10.7759/cureus.49159

**Published:** 2023-11-21

**Authors:** Khang Duy Ricky Le

**Affiliations:** 1 Department of General Surgical Specialties, The Royal Melbourne Hospital, Melbourne, AUS; 2 Department of Surgical Oncology, Peter MacCallum Cancer Centre, Melbourne, AUS; 3 Department of Medical Education, The University of Melbourne, Melbourne Medical School, Melbourne, AUS; 4 Department of Medicine, Deakin University, Geelong Clinical School, Geelong, AUS

**Keywords:** clinical education, medical simulation, simulation based teaching, medical student, medical education

## Abstract

A challenge for medical educators is to provide learning opportunities that allow students to develop technical and non-technical skills as set by the clearly defined learning objectives within their relevant institutions. This is particularly relevant in clinical education, which encompasses a vast majority of medical education. Specifically, clinical education is highly variable, with numerous distractions, interruptions and variability in learning experience and quality of clinical educators which often result in underprepared medical students by the time they transition into clinical practice. Simulation-based teaching (SBT) has been a key pedagogical approach that has been implemented into curriculum design to assist with addressing some of these educational challenges. However, their implementation is highly variable, and research into evidence-based best practice considerations in SBT design and implementation is fundamental to their success in medical student development.

A narrative review was performed following a computer-assisted search on electronic databases Medline, Embase and Google Scholar. Relevant papers that explored the role of SBT in medical education were considered for this review.

SBT is an important pedagogical approach to support the education of medical students. Their use has the benefit of providing a standardised and safe environment that mimics ‘real life’ as a means of allowing students to hone key skills with respect to clearly defined learning outcomes. The role of debriefing and feedback is crucial to the development of efficacious SBT programs, and therefore the upskilling and training of educators is a key aspect of evidence-based SBT design. Despite this, medical educators must be cognisant of the limitations of SBT. These include the cost and resources required to develop and implement SBT sessions, the effort and conceptualisation required to standardise and ensure these programs reflect real-life situations as well as the degree of training for facilitators to ensure they can best deliver and achieve learning outcomes and provide effective debriefing and feedback for students. Understanding the educational frameworks and the evidence-based best practice principles for SBT design and implementation is highly necessary for medical educators given the resource demands of SBT programs.

## Introduction and background

The core objective of medical educators is to produce well-rounded medical graduates. This task is complex, with many competing challenges in terms of acquisition of knowledge theory, mastery of clinical skills, development of clinical reasoning and building of non-technical skills (communication, patient advocacy, cultural awareness, research and education). Given the complexities, it is not unexpected that medical students often feel underprepared for their transition into junior doctors [1-3]. Central to this are feelings of anxiety and lack of confidence, which further hinder effective clinical practice [4]. This occurs in the background of variable hospital learning in which there are many competing interests in terms of clinician time, high amounts of distractions/interruptions and therefore disjointed and poorly standardised learning, debriefing and feedback opportunities [5-7].

Medical education has turned to more standardised experiential-learning pedagogies, specifically simulation-based teaching (SBT), to deliver effective medical education opportunities in light of these challenges. The legacy of this approach dates back at least to 17th-century France, where obstetric mannequins were utilised for the education and assessment of training doctors [8]. Moving forward to 21st-century medical education, SBT has shifted the way medical educators deliver lessons, with iterations including ward-based simulations, disaster scenarios, trauma simulations, clinical procedure simulations and simulated communication skills sessions. Simulations as an approach to education certainly have many advantages, including the opportunity to provide standardised learning situations that mimic real life, allowing students to practice and hone skills safely. Additionally, these opportunities also allow students to develop skills mastery, confidence, situational awareness and non-technical skills such as professionalism, patient advocacy and communication that are often poorly taught in the clinical environment. This is confounded by disadvantages attributed to cost, the ability to design relevant and engaging scenarios that fit with desired learning outcomes and the ability to mobilise resources adequately to effectively implement these sessions. Overall, the prevalence of these initiatives has been poorly characterised in the literature and varies on a case-by-case basis. Specifically, Huang et al. demonstrated that in North American teaching hospitals, simulation-based education varied depending on specialty including around 100% for nurses, 60% for respiratory therapists and <20% for social workers, dietitians and speech therapists [9]. In another study by Berg et al. in a North American institution, the prevalence of simulation for basic and advanced procedures was 46% and 23%, respectively [10]. Ultimately, these numbers vary depending on those who develop the curriculum as well as the resources available. Despite this, reliance on traditional methods of hospital-based teaching has led to challenges in the integration of SBT into existing medical curricula [11]. This is best exemplified by the heterogeneous ways in which SBT is facilitated at institutions across the world, often with variable results in methodology and efficacy. In a similar manner, there has been significant heterogeneity in the SBT programs in the literature, with no standard of best practice that is widely recognised or instituted. Furthermore, the complexities of imitating real-life scenarios in a standardised manner, in conjunction with meeting curriculum requirements and resources (both consumable and human), prompt medical educators to carefully consider the ways these opportunities are designed, implemented and integrated [12]. Deliberate consideration of evidence-based pedagogic theory, best-practice debriefing and feedback strategies and careful consideration of learning outcomes are essential to maximising learning benefits.

This review aims to evaluate the theories and outcomes from the contemporary medical education literature to provide a foundation for understanding evidence-based SBT approaches. It subsequently explores key principles and limitations of SBT with an additional emphasis on effective debriefing and feedback to identify best-practice strategies for the creation of SBT in the modern medical curriculum.

## Review

Methods

A narrative review was performed following a computer-assisted search of the Medline, Embase and Google Scholar databases. Relevant literature articles that explored the various aspects of SBT in medical student education including framework, theories, guidelines, best-practice principles, limitations and challenges were selectively considered in this narrative review.

Theories behind SBT

SBT lends itself to address many of the aforementioned challenges of hospital-based clinical education. The approach itself is rooted in various educational theories and frameworks that provide a versatile and useful adjunct to adult-based learning within medicine. Central to SBT and medical education in general are two main taxonomies of learning and thinking, Bloom’s taxonomy and Miller’s pyramid. These provide a framework to better understand the hierarchy of how students learn and develop their skills, particularly in the field of medicine.

Taxonomies of Thinking and Learning

Miller’s framework for assessing clinical competency in medical education is widely used across many faculties worldwide [11,13]. Relevant to SBT within this hierarchical model is the aim of educators to build upon clinical knowledge ('knows' and 'knows how') and transition to a phase of knowledge consolidation and action through supervised demonstration ('shows') followed by routine, competent and confident performance of the same skills ('does'). SBT offers students the opportunity to apply knowledge and skills to situations that mimic real-life situations yet still occur in a standardised and safe environment without the distractions and variability of the hospital setting. In this way, SBT facilitates higher-level integration and demonstration of knowledge for students. In a similar manner, SBT also lends itself to higher-order development of learning modalities as dictated by Bloom’s taxonomy [14]. Bloom’s taxonomy has traditionally been used in medical education to develop specific learning goals and objectives across the spectrum of active processes when applying knowledge. For educators who develop SBT lessons, a higher-order level of processing is required as dictated by Bloom’s taxonomy, namely that the educators must exhibit a more mature understanding of the content in order to create SBT scenarios and evaluate their function in terms of meeting learning outcomes and assessment that were originally planned.

Theories and Models of Learning

SBT can be considered a higher level of educational experience and assessment. As mentioned above, it requires both educators and learners to undertake a higher level of engagement and active processes towards the development of and engagement with learning, respectively. Two central theories underpin SBT well, namely adult learning theory and experiential learning theory.

Adult learning theory posits that learners are intrinsically motivated, independent, self-directed and self-regulated in their approach to learning from real-life situations [15]. This notion is highly relevant to SBT, as learners who are unique in their repertoire of competencies and skillsets are able to utilise SBT to identify specific learning outcomes, and therefore each experience of SBT is unique to the learner. In this way, learners can use a standardised scenario to achieve the learning outcomes set by educators in addition to exploring and developing learning outcomes to achieve for themselves. The role of SBT facilitators therefore becomes vastly important, as they must be versatile within the learning simulation to effectively guide learners through these dynamic learning opportunities. The process of judicious debriefing and feedback is therefore also highly necessary in SBT to assist in the development of adult learners. This is considered in Schon’s model of 'reflection-in-action', whereby learners in SBT are able to share and voice their thoughts whilst performing a simulation and reflect on the simulation experiences to further enhance their learning [16,17].

Moreover, central to SBT is experiential learning theory whereby learners gain education by directly engaging with scenarios of life [18]. The latter aspect of this definition is more synonymous with SBT as opposed to other types of non-hospital learning such as problem-based learning. This is because SBT offers learners the opportunity to be directly involved and participate in scenarios that mimic real life at higher levels of Miller’s pyramid [13]. One model that provides structure to experiential learning theory is Kolb’s experiential learning cycle [19]. In this model, learners cycle through four stages including a process of reflecting on the simulation experience ('reflective observation'), learning from the experience ('abstract conceptualisation'), planning an improved approach based on learning from the previous experience ('active experimentation') and doing the simulation experience ('concrete experience'). Repeated runs through these cycles in SBT offer learners the opportunity to hone their technical and non-technical skills and troubleshoot any challenges identified with respect to formal and intrinsic learning outcomes. Furthermore, observing students allows them to learn passively through vicarious activity.

Lastly, in an interesting review by McGaghie et al., the authors discussed SBT as a learning that is rooted in three main learning theory foundations: behavioural learning theory (BLT), constructivist learning theory (CLT) and social-cognitive learning theory (SCLT) [20]. In this framework, BLT focuses on behaviour change that is often noted during SBT as more knowledge is acquired through deliberate practice and feedback. CLT is often harder to define but conceptualises students as self-directed learners and emphasises their ability to use prior experiences (such as engagement with a prior simulation) to construct new perspectives, skills and knowledge. SCLT is perhaps more nebulous and frames learning as events that occur in simulated social settings and engagement in these simulated contexts contributes to knowledge and development. In all of these cases, the emphasis is not only on the self-regulation and self-direction of learners but also on the guidance of educators facilitating SBT. Critically, educators must provide guidance rather than information to allow students to, in a more autonomous fashion, achieve the desired learning outcomes. Educators must also structure the SBT experience in a way that allows learners to have opportunities to have new experiences, learn from them and engage them as required [21].

Key principles for the design of SBT sessions

Key elements of SBT design according to the literature begin initially with an overarching infrastructure or cycle in which events should be organised. One evidence-based mechanism for this is a cycle composed of five components: assess, design, develop, implement and evaluate [22,23]. In this cycle, the assess phase includes gathering information from multiple stakeholders including learners, accreditation, the course and the school to identify assessment needs and appropriate resources and methods. The design phase seeks to identify clear learning objectives and the educational context in which the SBT will fit, how it will be implemented and who will assist with this process. The develop phase seeks to identify a lesson plan and the necessary resources required to implement the SBT. The implement phase is where the finalised SBT is first implemented. There may be a pilot phase prior to the first official use to refine and improve the process. Lastly, the evaluate phase seeks to assess both learner performance and all processes of the SBT including the format in which it was implemented as a way for further improvement. Debriefing and feedback are critical in this area before the cycle can begin again [22,23].

A critical review of simulation-based medical education research over a seven-year period from 2003 to 2009 highlighted that best-practice SBT encompasses 11 key principles and best-practice features that educators should implement in their design of evidence-based and efficacious SBTs (Table 1) [11]. A 12th principle, known as the educational and professional context consideration, was also mentioned; however, its role in the design of the individual SBT is less relevant here [11].

**Table 1:** Eleven key features and best-practice principles of simulation-based education Adapted from McGaghie et al. (2016) [9]

Key principles
Feedback
Deliberate practice
Curriculum integration
Outcome measurement
Simulation fidelity
Skills acquisition and maintenance
Mastery learning
Transfer to practice
Team training
High-stakes testing
Instructor training

Feedback

The literature prioritises feedback as one of the most important variables to consider in the design of SBT [11]. Feedback is considered important in terms of the variety and source of feedback. Variety refers to the types of feedback received, such as through formative (where the task is performed for feedback with no assessment or progression repercussions) and summative (where the task is utilised for formal assessment) mechanisms [11]. Feedback should also come from multiple sources of feedback such as formal facilitators as well as colleagues and in some cases volunteer simulated patients. Lastly, a system of evidence-based debriefing is necessary to ensure learners are comfortable, supported and motivated for ongoing development. Therefore, adequate debriefing and feedback require both engagement by students and well-trained educators. New models that have approached this in simulation-based education are through the use of 'debriefing with good judgement.' Combining rigorous feedback models (e.g. Pendleton’s model) with curious enquiry urges students to reflect on their actions and educators to assist with reframing preconceived notions about this to identify learning goals moving forward.

Deliberate Practice

Deliberate practice refers to the intent of the learner in engaging with the SBT process. Deliberate practice principles include motivated and concentrated learners who are ready to engage in learning; well-defined learning outcomes to guide learning; appropriate level of difficulty for students; repetitive practice to enhance skills acquisition; rigorous measurement of outcomes; informative feedback to help with self-regulated learning and learner development; self-regulation and reflection from learners as a means for further engagement; evaluation process to ensure students achieve mastery; and a plan to advance to the next step to allow for continual development [24].

Curriculum Integration

Educators must recognise that SBT is not the silver bullet when it comes to educational opportunities but rather is one education modality that fits into the puzzle of other pedagogical approaches within the medical curriculum. Therefore, the types of simulation, assessments and learning outcomes developed as part of a SBT program must be considered in the landscape of all other learning opportunities, acting to complement these rather than override them.

Outcome Measurement

SBT approaches have the opportunity to provide both quantitative and qualitative outcomes, and these mixed methods are important considerations when it comes to outcome measurement. However, there are intrinsic biases in the reliability of such outcomes, particularly observational ratings of learner performance as well as learner responses. Identifying a valid and reliable method of outcome measurement is therefore fundamentally important to evaluate the effectiveness of the simulation session as well as the efficacy of the SBT on student development with respect to learning outcomes.

Simulation Fidelity

Effective SBT often requires matching the real-life task with technologies that allow the best possible mimic of this environment. Technological advancements have allowed quite realistic situations to be manifested, such as through models for procedural skills training, high-fidelity simulated mannequins with real-time monitoring of vital signs (e.g. SimMan) for patient interactions and nowadays even virtual and augmented reality.

Skills Acquisition and Maintenance

One of the benefits of SBT is that it allows repetitive practice of technical skills that otherwise in real-life clinical contexts may be haphazard and disjointed. These skills may include surgical skills, procedural skills or approaches to management (e.g. primary survey in trauma, advanced cardiac life support and cardiopulmonary resuscitation). Repetition is a key feature of SBT that allows efficacious skills acquisition. SBT programs should consider repetitive practice over multiple sessions (on top of repetition within the same session) as evidence suggests failure to do so leads to skills decay without further practice [25].

Mastery Learning

Mastery learning refers to rigorous approaches to competency-based education that occur concurrently with deliberate practice. Key features of this include baseline testing as a starting foundation; having clearly defined learning objectives to ensure learning activities meet the demands of the curriculum; engagement in skills activities; having established and clear minimum standards by which assessment is based to ensure adequate competency is met; formative assessment as a means to gauge unit completion; and a plan for advancement to the next unit to ensure continued practice and mastery is achieved [24].

Transfer to Practice

This refers to translatable skills that can be used in real-life clinical settings which are considered a key aspect of the considerations of learning outcomes and activities within each SBT program [11]. In essence, the skills or learning outcomes for the SBT should be relevant to the clinical practice of the learner [11].

Team Training

A key aspect of SBT is the opportunity to engage in simulated multidisciplinary environments to practice non-technical skills in teamwork, collaboration and communication [11]. Educators involved in the design of SBT programs should consider effective planning, implementation and evaluation of teamwork specific to healthcare [11].

High-Stakes Testing

Standardisation, reproducibility and fidelity of the simulation of SBT, as well as the quality of the educational experience in mastering learning outcomes and skills, lend themselves to a formative and summative evaluation of competencies [11]. Furthermore, given the opportunity for SBT programs to also assess non-technical skills including professionalism and communication skills on top of procedural skills, SBT offers itself as an effective tool for global medical assessments [11].

Instructor Training

Given the complexity of the theory and implementation of SBT and its reliance on the facilitator as a guide for learners in their professional development, adequate instructor training is imperative. It has been suggested that clinical experience or seniority alone is not a proxy for effective training in the delivery of SBT, debriefing and feedback necessary to allow maximal learning to be achieved from these sessions [11]. In addition to this, educators must also consider the volunteer patient as an instructor, and appropriate training for this cohort is necessary to ensure SBT programs are delivered effectively.

Outcomes of SBT

Simulation is a cornerstone of teaching in many high-reliability industries such as the aviation industry where there is an exceptionally high standard of quality and safety. Drawing from these learnings, the question then is whether similar improvements in skills training and therefore safety in healthcare can be achieved with the assistance of SBT. SBT has been associated with improvements in both technical and non-technical skills for medical students. Perhaps the most widely considered aspect of this is procedural skills education. Randomised controlled studies have demonstrated that following an appropriately designed SBT, learners demonstrated improvement in procedural skills including lumbar puncture, central venous access and bladder catheterisation [26-29]. SBT has subsequently found a niche in various programs to assist in learner education in this space including basic life support, advanced life support, ultrasound-guided cannulation courses and advanced trauma life support (ATLS). This also demonstrates the key role of simulation in not just medical student education but also the ongoing relevance and importance of simulation as a pedagogical approach to education for more advanced medical training. It is important to recognise that repetition is fundamentally important to solidify the skills acquisition obtained from SBT, as failure to do so will lead to a period of skills decay following the completion of the learning session. Educators therefore must consider the role of the SBT in the global curriculum for learning and future opportunities for ongoing skills maintenance outside of the SBT [30,31].

More variable outcomes have been demonstrated in studies that evaluate the progression of non-technical skills following SBT programs. These skills, which include task management, teamwork, situational awareness and decision-making, are often more difficult to assess, with an element of subjective judgement from those tasked to assess them [32]. In a study by Morgan et al., an in-house scoring system to measure non-technical skills demonstrated minimal improvement [32]. The researchers hypothesised that reasons for this may include insufficient intervention (SBT) to allow for a change in behaviours as well as the possibility that the study cohort (experienced anaesthetists) may already have a heightened level of these skills that meaningful improvement is less likely to be identified. Despite these outcomes, other studies in different settings have demonstrated significant improvement following SBT in non-technical skills in areas such as teamwork in trauma teams and leadership and task management in neonatal resuscitation situations [33,34].

Overall, it is important for educators to recognise that outcomes associated with SBT, in terms of both student performance and the SBT itself, are poorly characterised. In particular, studies that evaluate SBT programs generally utilise mixed methods. With respect to SBT evaluation, these tend to be in the form of focus groups, semi-structured interviews and feedback, which lends itself to bias in the ways SBT programs are evaluated on multiple aspects of their performance including relevance, realism, ability to meet learning objectives, ability to develop key technical skills and overall effectiveness. Additionally, when it comes to student outcomes, evaluations of student progress are often in the form of direct quantitative surveys post-simulation which can include Likert scales of confidence or indirectly through their performance in other formal clinical assessments such as objective structured clinical exams. These outcomes are also further confounded by additional educational pedagogies in medical education, including hospital-based learning, problem- or case-based learning, lectures, tutorials and individual self-regulated learning. This makes it difficult for medical educators to evaluate the direct effect of their SBT programs and understand the impact that these programs may have on specific student clinical skills as well as patient outcomes when students transition to clinical practice. The challenge therefore for medical educators and researchers is to identify robust methods of evaluating their programs to better understand their effect on student learning and improvement subsequently on patient outcomes [27].

Limitations of SBT

Perhaps one of the largest barriers to effective SBT is the cost and resources required for its development and implementation. Specifically, the time to create realistic scenarios and settings, which may involve utilising various technologies such as simulated mannequins and monitors, the need for consumables and the training of facilitators are essential yet resource intensive in the process of SBT design [35]. Furthermore, multiple cycles of quality improvement may be required before the first iterations of SBT are implemented for learners, further increasing the demand for time and resources. The use of sustainable tools such as SimMan, although initially expensive but versatile in application, may be an approach to alleviate these costs. Furthermore, the advent of generative artificial intelligence tools offers exciting potential to improve efficiency, time and creative potential in designing and drafting SBT scenarios and programs. In many jurisdictions, medical schools will also vary in their resources, and therefore the SBT that is developed in each setting may differ in resources and therefore in the way it can be implemented.

Additionally, adequate mechanisms to evaluate performance are fundamental to evaluating the SBT that has been designed. When these systems of evaluation are not effectively designed, it may be difficult for both educators and learners to adequately identify specific learning outcomes and improvements in technical and non-technical skills, therefore impairing the ability to further refine the simulation process [31,33]. These systems therefore must be refined, validated and undergo judicious development to ensure they are suitable for the specific aims of the SBT that is to be implemented.

Lastly, given the heterogeneity and unpredictability of the human hospital environment, a significant limitation of simulations lies in their ability to reflect these environments adequately. Certainly, a benefit of SBT is the ability to produce standardised and realistic representations of this for safe learning of skills; however, doing so also has the limitation of restricting the processes, reactions and learning opportunities that can be gained. If there is a reliance on SBT for learning, this may hinder the progression of the dynamic decision-making skills that are required within clinical practice. It is important for facilitators and learners to recognise that the SBT is one of the many pedagogical approaches within the medical curriculum to support global development and not rely on SBT to accurately represent all the potential circumstances that may arise in the hospital setting.

Debriefing and feedback

Central to experiential learning theory which guides SBT is the role of reflection to aid learning, identify new learning outcomes and guide future development [19]. For example, as part of deliberate practice, feedback allows learners to identify specific goals in the immediate setting as a mechanism for skills improvement and acquisition in real-time [11]. Furthermore, the process of debriefing and feedback following the completion of the simulation by the learner allows students to gain further insight into how they may self-regulate and approach future learning with respect to both self-identified and curriculum-identified learning objectives [11]. The debriefing and feedback process also allows educators to gain insight into avenues to explore in the quality improvement of the SBT programs that they have designed and/or implemented [22,23]. Such methods to do this have traditionally relied on mixed methods, including quantitative improvement of learners in the relevant defined learning outcomes as well as qualitative feedback through surveys and focus groups.

One way this process is utilised in real-life settings is through the provision of trauma management education in the ATLS course. In this course, debriefing and feedback are provided on multiple instances allowing for feedback to learners from facilitators, feedback to learners from other learners and feedback to the faculty and the course itself from learners [11]. Models of feedback however are more variable and can range from formal to informal mechanisms. One way that is utilised within the ATLS program is through the Pendleton model of feedback where facilitators first ask the learner what went well, then tell the learner what went well, followed by asking and telling the learner what could be improved on [36]. More contemporary approaches have been built upon this model through more effective debriefing strategies. One method for this is through curious enquiry utilising a debriefing with a good judgement model [37]. In this model, prompts similar to the Pendleton model are utilised with the addition of inquisitive questioning to prompt self-regulated reflection, learning and identification of learning goals from the student. This model has proven to be an effective way of providing feedback as it allows learners to engage in active reflection, intrinsic motivation and also improvement in their own ability to approach debriefing and feedback [38]. Regardless of the format, a key aspect of debriefing and feedback is the intimate link between simulation, debriefing, and feedback that is fundamental to improved learning for students and the quality development of SBT programs.

Future directions

Advancement in simulated technologies, such as high-fidelity simulated mannequins (e.g. SimMan), has allowed institutions to provide simulated medical scenarios with changes to vital signs, haptic feedback (e.g. breath sounds, pulses) during clinical examination and monitoring of clinical skills (e.g. quality of chest compression) in real-time. These technologies allow educators access to a greater degree of educational data that may improve their ability to evaluate SBT programs with a more rigorous methodology. These technologies however remain highly scarce, with many institutions varying in their capacity to acquire and maintain these tools or justifying the cost compared to other alternatives such as volunteer patients.

Additionally, with the advent of widely accessible open-access generative artificial intelligence (AI) tools, the capacity for educators to develop SBT programs has significantly increased. For educators, the time required to develop simulation plans and scenarios is time consuming. Generative AI tools such as ChatGPT and Google Bard could potentially be leveraged for this purpose. Despite this, their use must be exercised cautiously. For example, Han et al. demonstrated that ChatGPT failed to adequately identify learning outcomes and appropriate evidence-based material when tasked to generate a lesson on hyperlipidaemia [39]. The use of these technologies therefore warrants judicious auditing of their outputs to ensure that the SBT program meets the demands and curriculum standards set out by educators who have been tasked to design them.

Lastly, given the resource-intensive nature of SBT programs, there remains a strong consideration of how to best deliver these best-practice methods universally. Future research should consider ways to improve costs and develop tools that can be repurposed and leveraged in multiple settings and are widely accessible to ensure there is equitable access to learning opportunities for all medical students.

## Conclusions

This review has provided a comprehensive exploration of the foundational research that underpins the quality and effective design and implementation of SBT. In particular, this review highlights the key principles of SBT design and the debriefing and feedback process as crucial aspects of SBT implementation. By synthesising the insights from the literature, this review better equips educators for the design, implementation and improvement of SBT programs in the landscape of the modern medical curriculum.
